# Supplementary material for: Land conversion to agriculture induces taxonomic homogenization of soil microbial communities globally
Source: Nat Commun. 2024 Apr 29;15:3624. doi: 10.1038/s41467-024-47348-8 (PMC11058813; doi:10.1038/s41467-024-47348-8)
Supplement: Supplementary file 1 — Supplementary Information [file 41467_2024_47348_MOESM1_ESM.pdf]

Supplementary information for

Land conversion to agriculture induces taxonomic homogenization of soil microbial communities globally

Content of figure and table

Supplementary Fig. 1-13

Supplementary Table 1-4

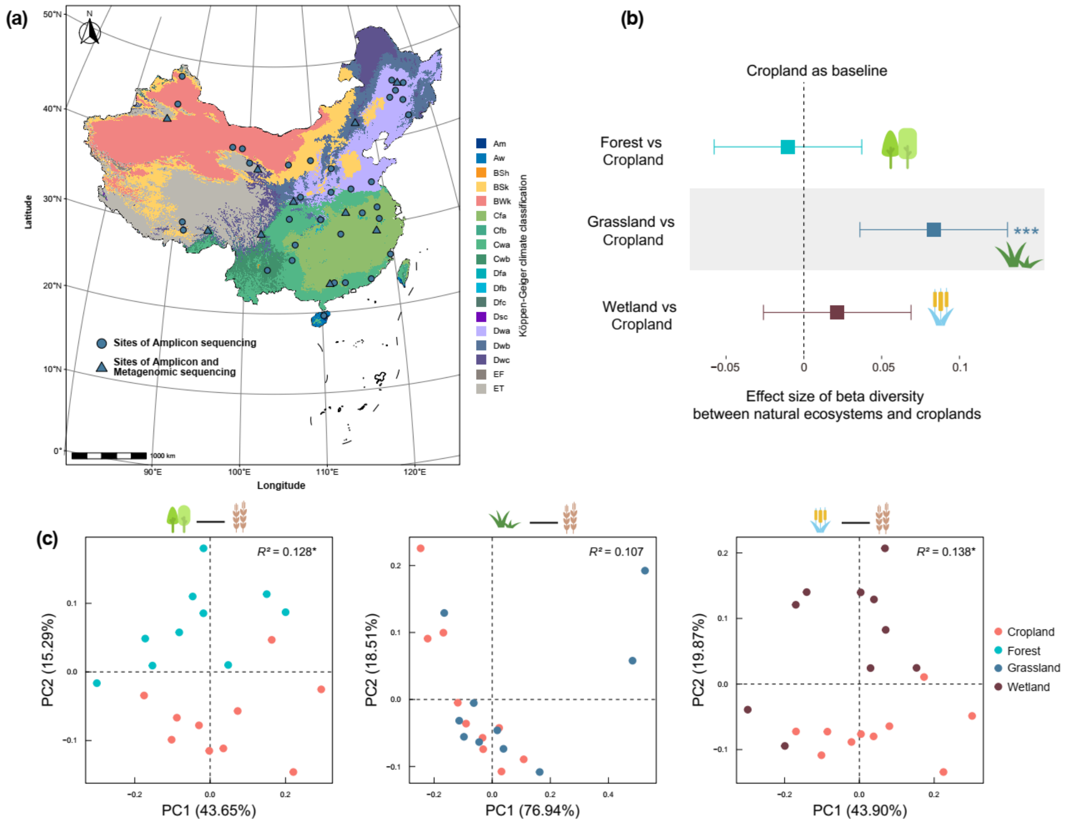

Supplementary Fig. 1 Effects of agricultural land use on functional composition annotated with COG. a, Map showing 10 regions in covering croplands and adjacent forest, grasslands, and wetlands from metagenomes. **b,** Effect sizes of natural ecosystems on β-diversity in functional composition annotated with COG relative to croplands. Data are presented as mean ± s.e.m. of the estimated effect sizes. Statistical significance is based on F-test; $***p < 0.001$. **c,** Principal coordinate analyses of Bray–Curtis distances showing dissimilarities among functional composition annotated with COG between croplands and natural ecosystems, including forests, grasslands, and wetlands. Communities differed among ecosystem types using PERMANOVA: $*p < 0.05$. This figure is similar to Fig. 1h, where the functional composition annotated with KEGG. N = 10 biologically independent samples for each ecosystems.

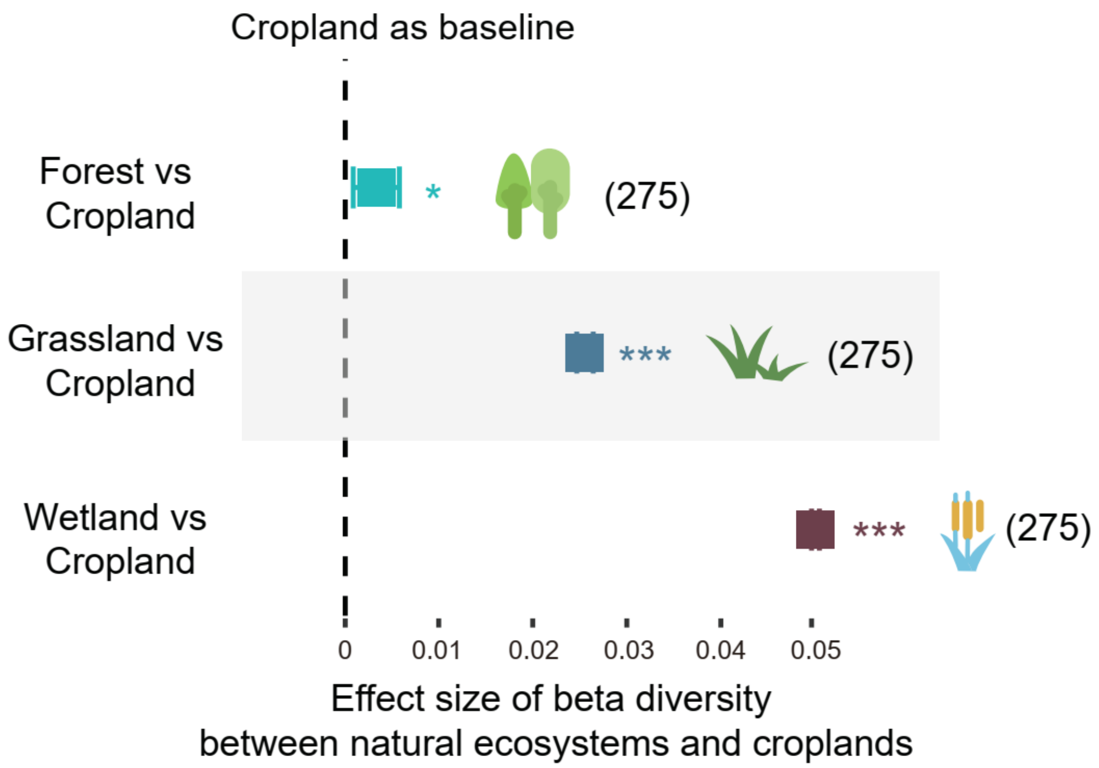

Supplementary Fig. 2 Effect sizes of natural ecosystems impacts on β-diversity in taxonomic composition with the same number of plots for grassland, forest, and wetland. Data are presented as mean ± s.e.m. of the estimated effect sizes. Sample size is showed by number of data pairs for each group. Statistical significance is based on *F*-test; ***$p < 0.001$, **$p < 0.01$, *$p < 0.05$. This figure is similar to Fig. 1d with the only difference that here we standardized the number of samples analyzed to 275 per cropland-natural ecosystem comparison.

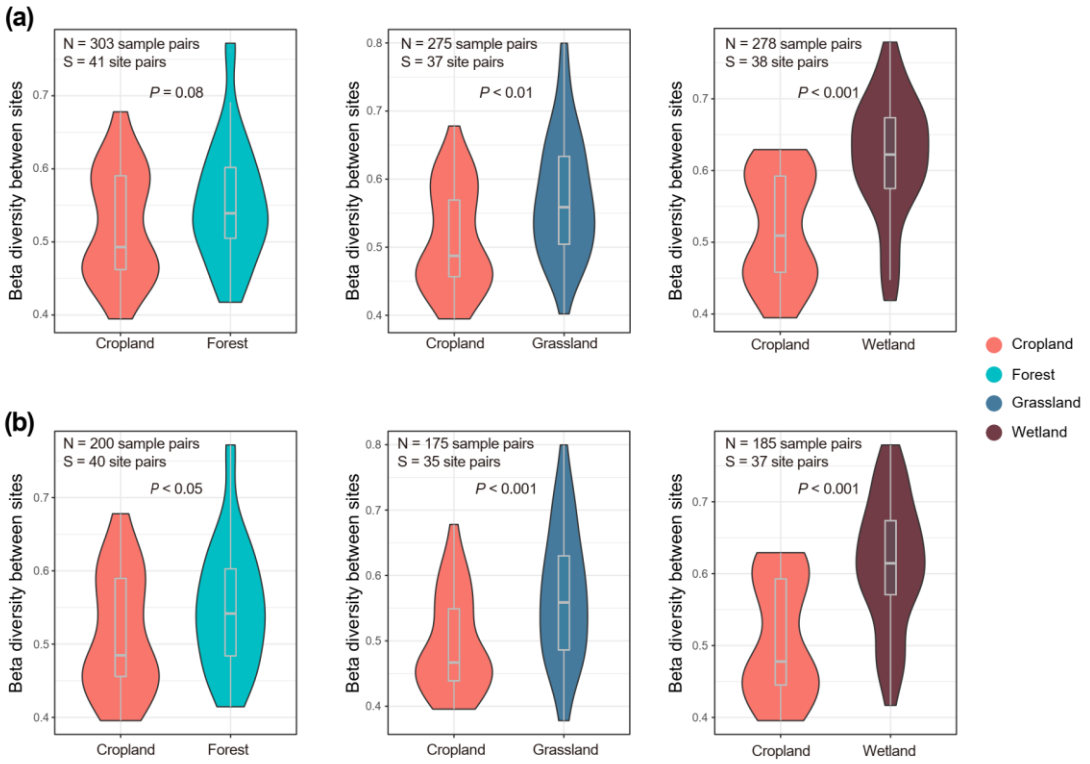

Supplementary Fig. 3 Response of community dissimilarity (beta diversity) to agricultural impacts between sites between cropland and forest, grassland, and wetland. (**a**) All 1,185 samples were included in the analysis. The total number of each site is shown in Table S2. (**b**) To exclude the confounding effect of sample size, five randomly selected samples from each ecosystem in a given site were included in the analysis. Statistical significance based on Wilcoxon test.

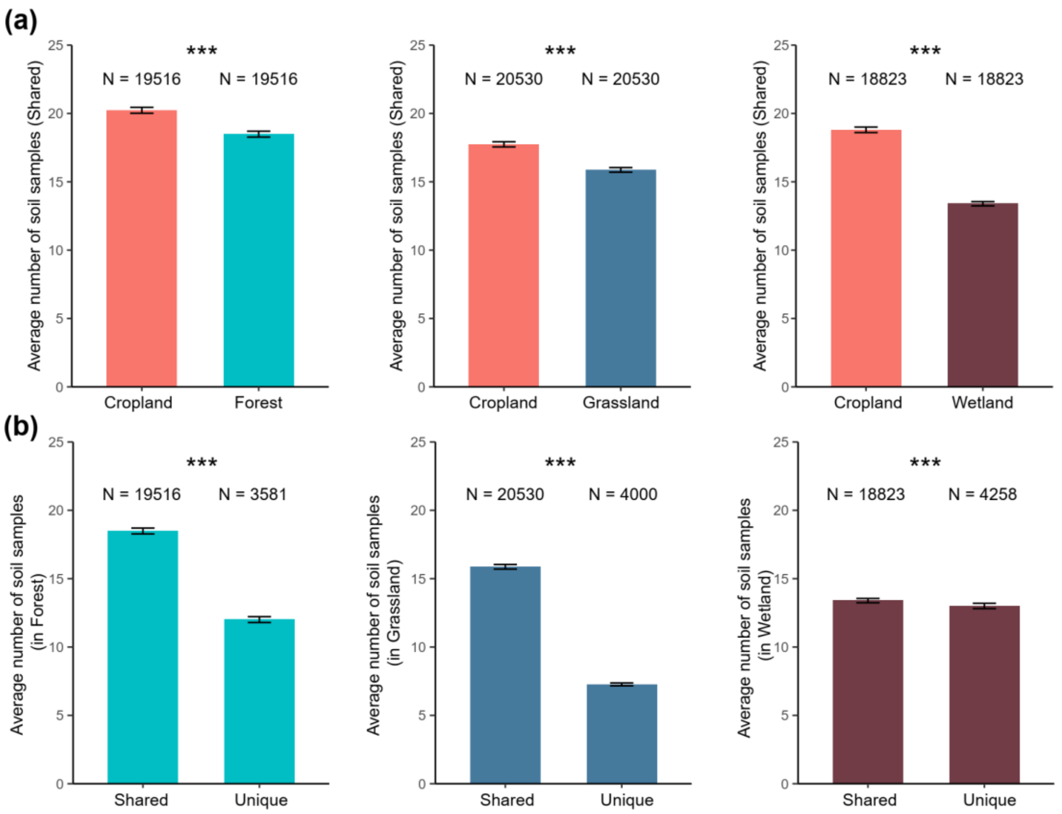

Supplementary Fig. 4 Distribution of microbial ASVs across soil samples. (**a**) Distribution in natural ecosystems and croplands of ASVs shared between natural ecosystems and croplands. (**b**) Distribution in natural ecosystems of ASVs unique to natural ecosystems and ASVs shared between natural ecosystems and croplands. We calculated the occupation number of soil samples for each shared ASVs in natural ecosystems and croplands (**a**), and for each unique ASVs in natural ecosystems (**b**). N indicates the number of ASVs used in the related analyses. Statistical significance based on Wilcoxon test is indicated by: $***p < 0.001$.

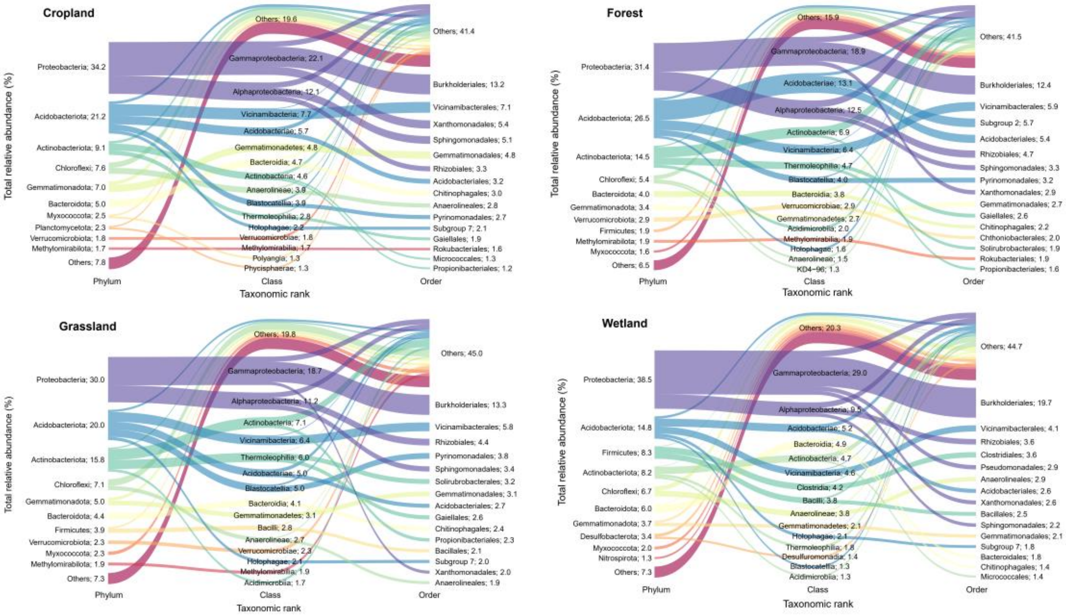

Supplementary Fig. 5 Community composition in cropland soils and natural ecosystems. Alluvial plot showing the total relative abundance distribution of different ecosystems across different taxonomic ranks (phylum, class, and order). The height of the sections displays the relative abundance (indicated in the text; the total is 100%).

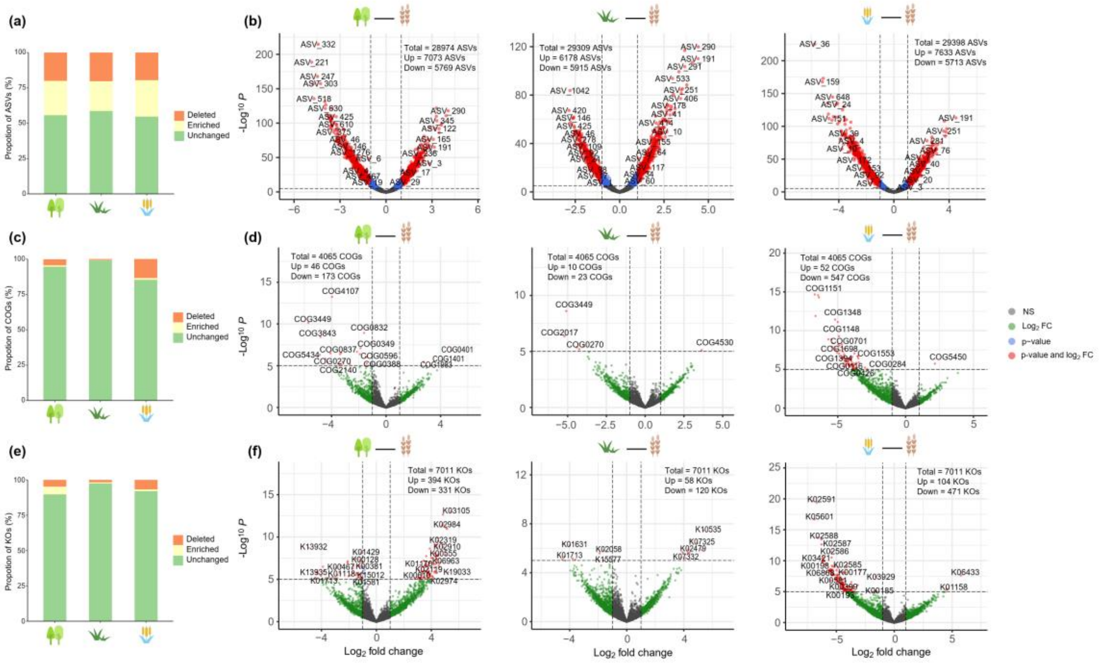

Supplementary Fig. 6 Effects of agricultural impacts on microbial phylotypes and funtional annotation. Colours of bar plots show the proportions of phylotypes (**a**), functional gene annotation assigned to COG (**c**) and KEGG (**e**) that significantly increased, decreased and unchanged under agricultural impacts. The volcano plot illustrating the enrichment and depletion patterns of microbial phylotypes (**b**), functional gene annotation assigned to COG (**d**) and KEGG (**g**) in croplands compared with natural ecosystems.

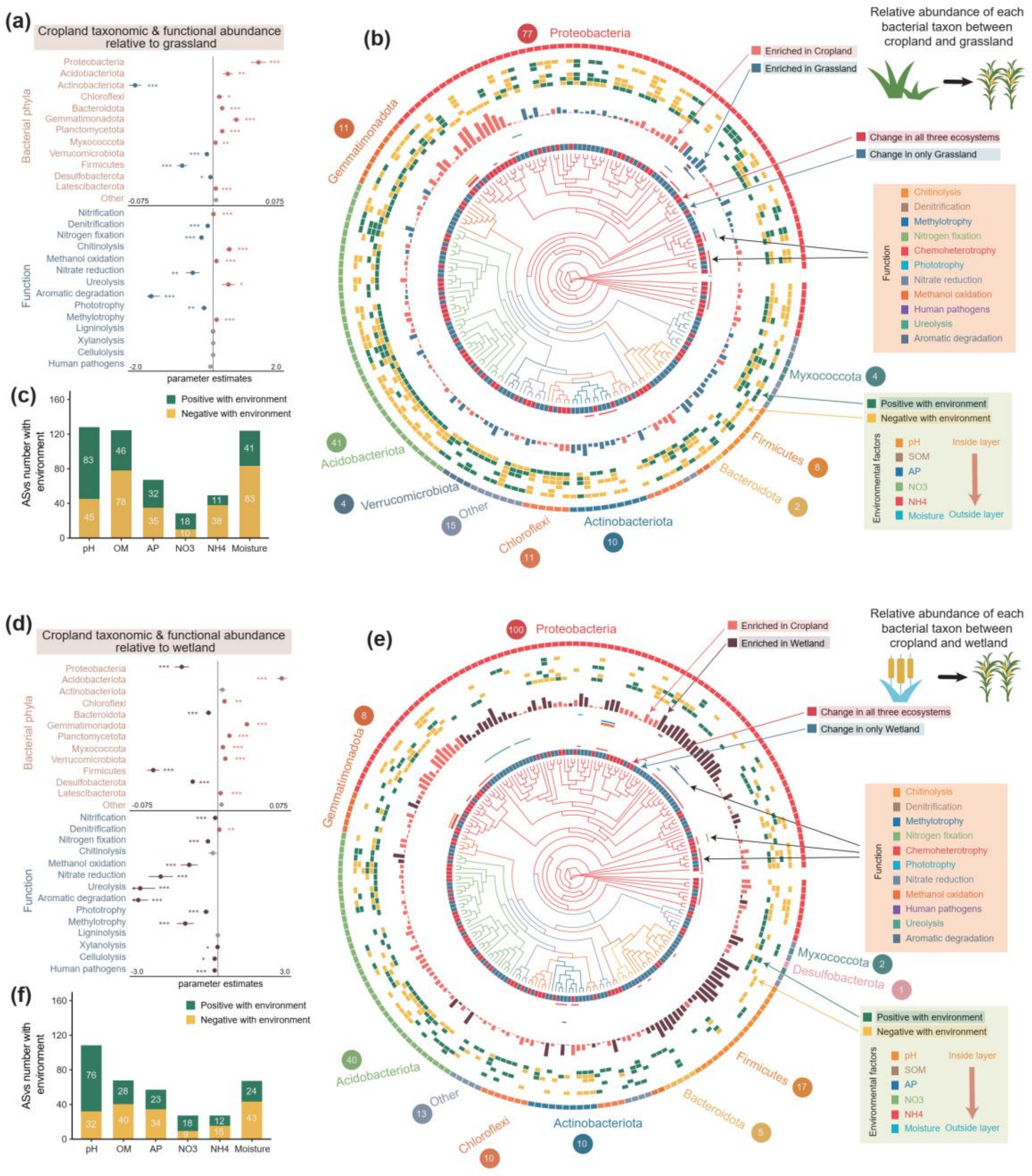

Supplementary Fig. 7 Effects of agricultural conversion on different microbial taxa compared with grasslands and wetlands at continental scale. a and c, Effect sizes of agricultural impacts on the relative abundance of major microbial taxonomic groups and functional groups as classified by FAPROTAX compared with grasslands (**a**) and wetlands (**d**). A total of 275 grassland-cropland pairs (**a**), and 278 wetland-cropland pairs (**d**) were compared. The estimated effect sizes are regression coefficients based on the linear mixed-effects models. Data are presented as mean ± s.e.m. of the estimated effect sizes. Statistical significance is based on Wald type II χ^2 tests; $***p < 0.001$, $**p < 0.01$, $*p < 0.05$. Non-significant changes are denoted by grey dots.

b and d, The phylogenetic relationships of individual microbial phylotypes with a significant response ($p < 0.05$) based on Wald test using DESeq2 to agricultural impacts and with average relative abundance > 0.05% among croplands and grasslands (**b**) and wetlands (**d**). Colours of the branches in the first and sixth rings correspond to individual phyla. Colours of the second ring represent phylotypes with significant increase or decrease under agricultural impacts. Colored blocks of the third ring represent the performance of a particular function by at least one phylotypes. The bars of the fourth ring represent the positive and negative effect sizes of agricultural impacts on relative abundances of phylotypes. Colored blocks of the fifth ring represent Spearman's correlation between the relative abundance of phylotypes and soil physicochemical properties (from inner to outer rings: pH, SOM, AP, NH_4 , NO_3 and moisture). **c and f,** Bar plots show the number of phylotypes that were significantly correlated with specific soil physicochemical properties among croplands and grasslands (**c**) and wetlands (**f**). pH, soil pH; SOM, soil organic matter; AP, available phosphorus; NH_4, ammonium nitrogen; NO_3, nitrate nitrogen.

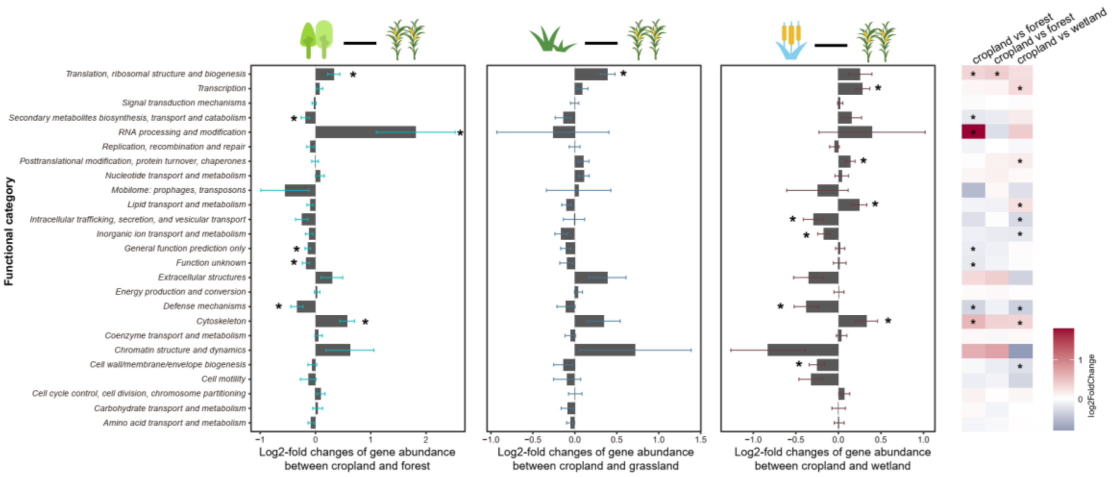

Supplementary Fig. 8 General impact of agricultural impacts on microbial functions. Log2-fold changes (LFC) in functional genes annotated with COG aggregated over functional categories in croplands relative to forests, grasslands and wetlands. Asterisk indicate a statitistical significant (p < 0.05) difference between croplands and natural ecosystems based on Wald test using DESeq2. Each bars represent the LFC ± s.e.m. N = 10 biologically independent samples for each ecosystems.

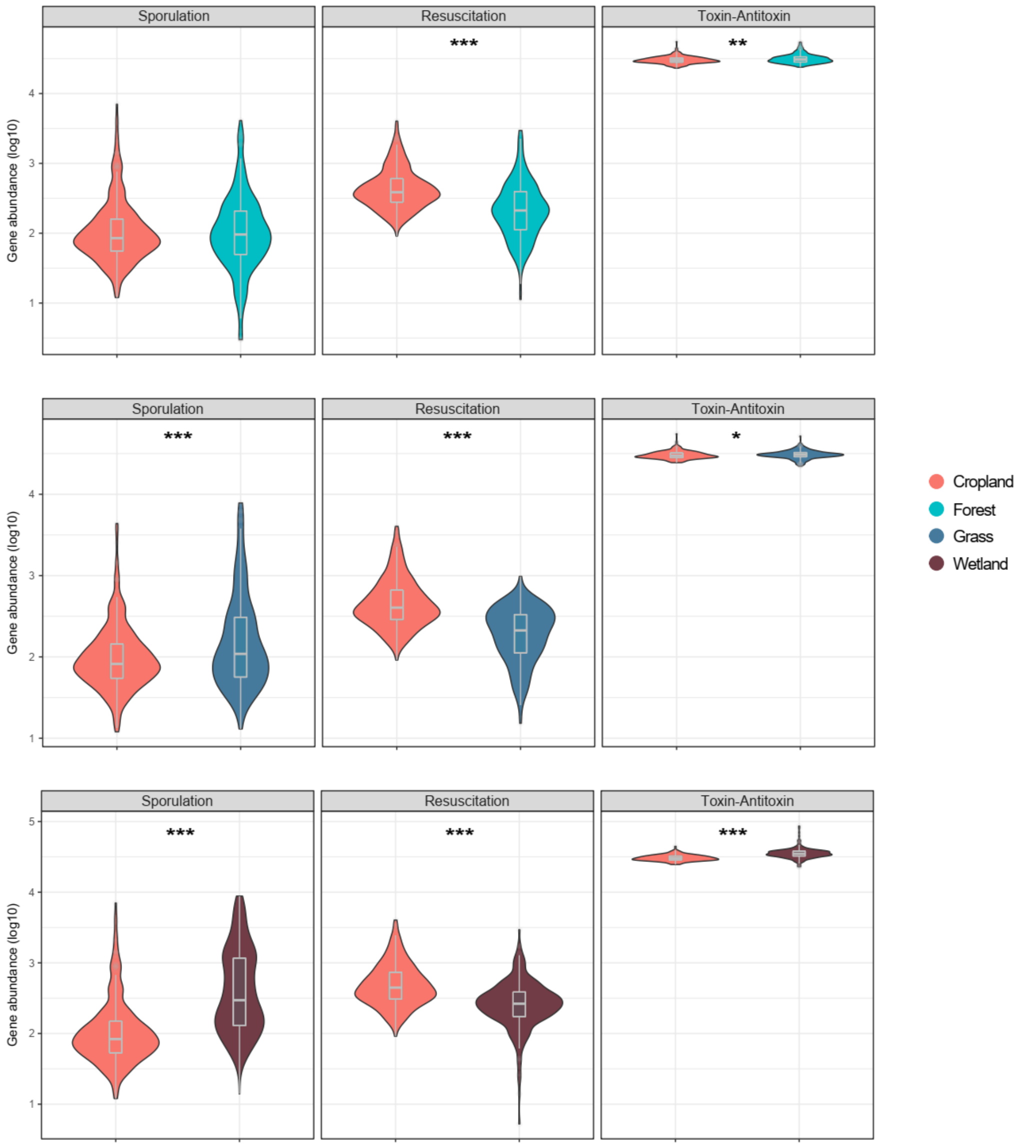

Supplementary Fig. 9 General impact of agricultural impacts on genes underlying dormancy strategies. Dormancy genes include sporulation factors, toxin–antitoxin systems, and resuscitation-promoting factors identified by PICRUSt2. Statistical significance based on Wilcoxon test is indicated by: ***$p < 0.001$, **$p < 0.01$, *$p < 0.05$. A total of 303 forest-cropland pairs, 275 grassland-cropland pairs, and 278 wetland-cropland pairs were compared.

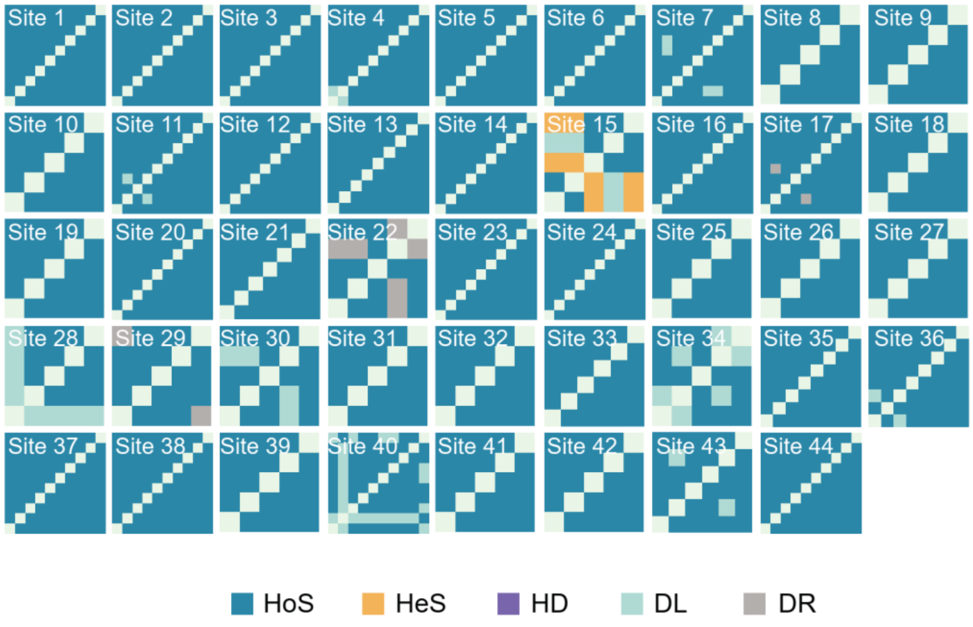

Supplementary Fig. 10 Community assembly processes between paired samples in 44 sites of croplands. Colored blocks represent different assembly processes. Hos, homogeneous selection; HeS, heterogeneous selection; DL, dispersal limitation; HD, homogenizing dispersal; DR, drift and others.

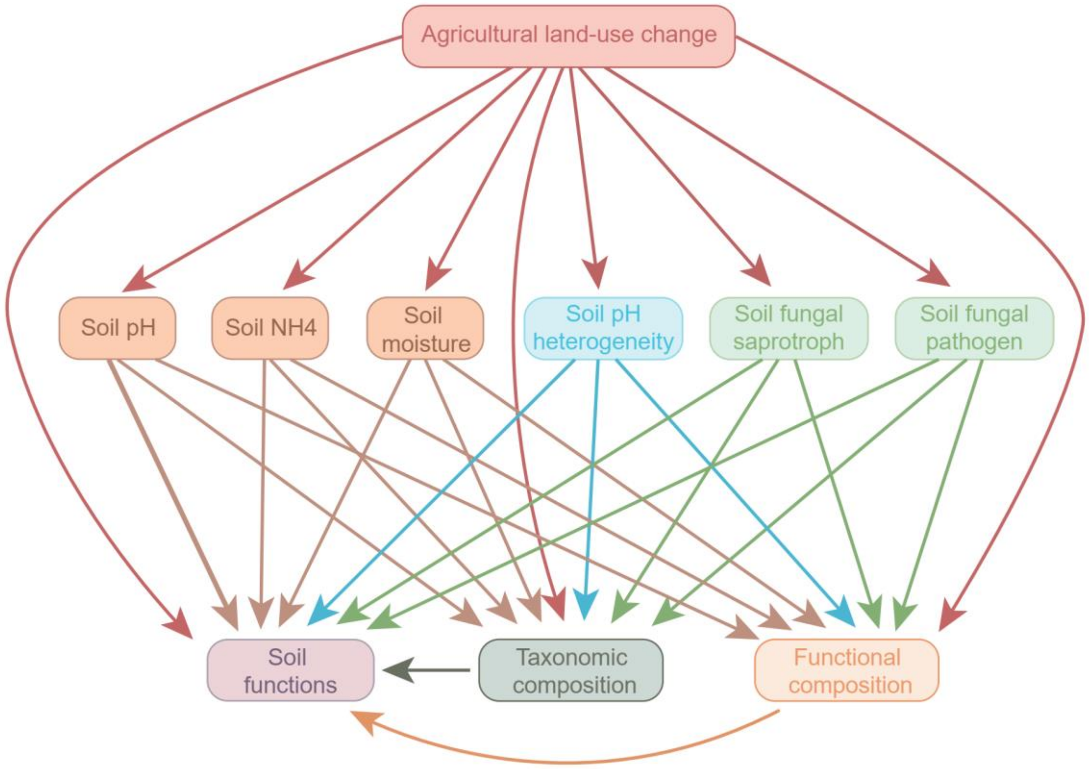

Supplementary Fig. 11 A priori conceptual model on the relationships between agricultural land-use, environmental variables, microbial taxonomic and functional composition, and soil function.

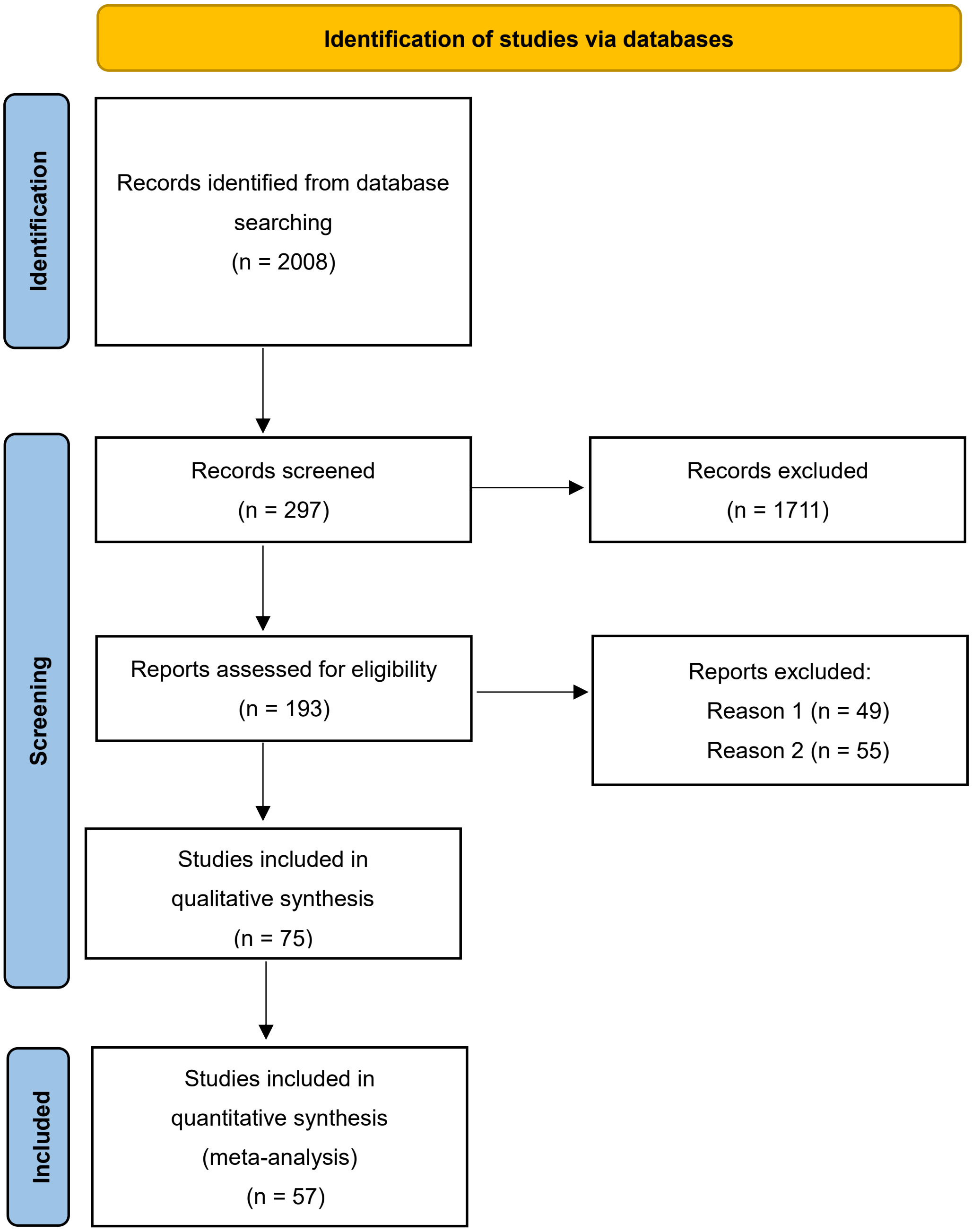

Supplementary Fig. 12 PRISMA flow diagram for the studies selected and included in the systematic review.

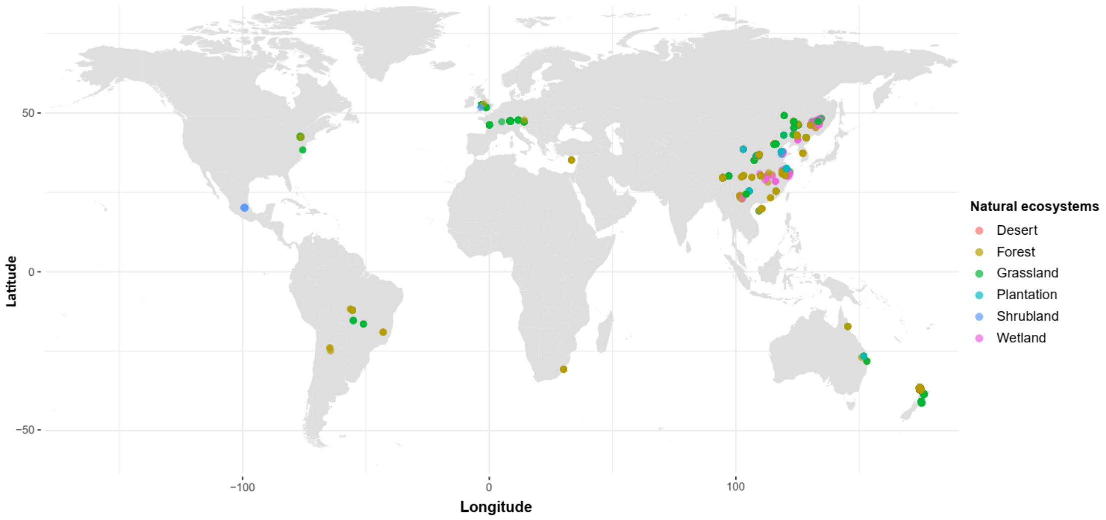

Supplementary Fig. 13 Geographical distribution of the natural ecosystems samples from global-scale meta-analysis.

Supplementary Table 1. The sampling locations of 44 sites. A tick indicates that the site covers the corresponding ecosystem types.

Site	City	Longitude	Latitude	Cropland	Forest	Grassland	Wetland	Soil type
1	Yulin	109.52	38.53	√	√	√	-	Skeletol primitive soils
2	Shangqiu	115.21	34.68	√	√	√	√	semi-hydromorphic soil
3	Rizhao	118.35	35.19	√	√	√	√	Hydromorphic soils
4	Yinchuan	106.11	38.12	√	√	√	√	Anthrosols
5	Haibei	101.43	37.52	√	√	√	√	Alpine soil
6	Dangxiong	91.06	30.47	√	-	√	√	Hydromorphic soils
7	Aertai	87.31	47.79	√	√	√	√	Arid soils
8	Nanjing	118.63	32.08	√	√	√	√	Anthrosols
9	Liuan	116.44	31.63	√	√	√	√	semi-hydromorphic soil
10	Luoyang	112.25	34.53	√	√	√	-	semi-hydromorphic soil
11	Zhaoqing	111.79	23.52	√	√	√	√	Anthrosols
12	Luding	102.13	29.65	√	√	√	√	Luvisols
13	Taigu	112.58	37.38	√	√	√		Semi-Luvisols
14	Zhangye	100.15	38.31	√	√	√	√	Saline soils
15	Yumeng	97.39	40.10	√	√	√	√	Anthrosols
16	Songyuan1	123.75	44.75	√	√	√	-	Caliche Soils
17	Guiyang	106.34	26.52	√	√	√	√	Skeletol primitive soils
18	Guangzhou	113.22	23.42	√	-	√	√	Anthrosols
19	Yueyang	113.13	29.37	√	√	√	√	Ferralisols
20	Linzhi	94.73	29.72	√	√	√	√	Luvisols
21	Liuba	106.73	33.62	√	√	√	√	Luvisols
22	Wujiaqu	87.55	44.41	√	√	-	-	semi-hydromorphic soil
23	Taibai	107.76	34.19	√	√	√	√	Semi-Luvisols
24	Kuerle	86.30	42.40	√	√	√	√	Skeletol primitive soils
25	Songyuan2	124.99	45.40	√	√	√	√	semi-hydromorphic soil
26	Changchun	125.83	44.12	√	√	√	√	Skeletol primitive soils
27	Xuancheng	118.70	30.67	√	√	√	√	Ferralisols
28	Daqing	124.81	46.69	√	√	√	√	Caliche Soils
29	Haerbin	126.65	46.06	√	√	-	√	semi-hydromorphic soil
30	Suihua	125.62	46.22	√	√	√	√	Caliche Soils
31	Chengde	117.22	42.46	√	√	√	√	semi-hydromorphic soil

32	Chaozhou	116.65	23.55	√	√	√	√	Anthrosols
33	Songming	103.04	25.33	√	√	√	√	Anthrosols
34	Fuzhou	119.56	26.19	√	√	√	√	Anthrosols
35	Xinyang	114.09	31.85	√	√	√	√	semi-hydromorphic soil
36	Tonghua	126.10	42.18	√	√	-	√	Semi-Luvisols
37	Jiuquan	98.92	40.00	√	√	-	-	Anthrosols
38	Yichang	110.47	31.32	√	√	-	√	Luvisols
39	Langzhong	106.07	31.54	√	√	√	√	Saline soils
40	Kaihua	118.11	29.25	√	√	√	√	Ferralisols
41	Wuzhou	111.21	23.39	√	√	√	√	Ferralisols
42	Tongzi	106.80	28.39	√	√	√	√	Skeletol primitive soils
43	Haikou	110.21	19.59	√	√		√	Ferralisols
44	Lasa	91.33	29.50	√	-	√	√	Alpine soil

Supplementary Table 2. A total of 1185 soil samples including 856 paired soils across sites and ecosystems at continental scale. Number indicates the number of amplicon samples in each ecosystem in given a site. NA indicates no samples in ecosystem. A tick indicates that metagenomic data are measured in given a site.

Site	City	Cropland	Forest	Grassland	Wetland	Metagenome
1	Yulin	10	10	10	NA	
2	Shangqiu	10	10	10	10	
3	Rizhao	10	10	10	10	
4	Yinchuan	10	10	10	10	
5	Haibei	10	10	10	10	√
6	Dangxiong	10	NA	10	10	
7	Aertai	10	10	10	10	
8	Nanjing	5	5	5	5	
9	Liuan	5	5	5	5	
10	Luoyang	5	5	5	NA	
11	Zhaoqing	10	10	10	10	
12	Luding	10	10	10	10	√
13	Taigu	9	9	9	NA	
14	Zhangye	10	10	10	10	
15	Yumeng	5	4	5	5	
16	Songyuan1	10	10	10	NA	
17	Guiyang	10	10	10	10	
18	Guangzhou	5	NA	5	5	
19	Yueyang	5	5	5	5	
20	Linzhi	10	10	10	10	√
21	Liuba	7	7	7	7	√
22	Wujiaqu	5	5	NA	NA	
23	Taibai	10	10	10	10	
24	Kuerle	10	10	10	10	√
25	Songyuan2	5	5	5	5	
26	Changchun	5	5	NA	5	
27	Xuancheng	5	5	5	5	
28	Daqing	5	5	5	5	
29	Haerbin	5	5	NA	5	

30	Suihua	5	5	5	5	√
31	Chengde	5	5	5	5	√
32	Chaozhou	5	5	5	5	
33	Songming	6	6	5	5	
34	Fuzhou	5	5	5	4	
35	Xinyang	8	8	8	8	√
36	Tonghua	8	8	NA	8	
37	Jiuquan	10	10	NA	NA	
38	Yichang	10	10	NA	10	
39	Langzhong	5	5	5	5	
40	Kaihua	10	10	10	10	√
41	Wuzhou	5	5	3	5	√
42	Tongzi	5	5	3	5	
43	Haikou	6	6	NA	6	
44	Lasa	10	NA	10	10	

Supplementary Table 3. Permutational multivariate analysis of variance (PERMANOVA) comparing cropland and natural ecosystems plots (forest, grassland and wetland) by each site.

	Forest			Grassland			Wetland		
Site	R^2	P	ASVs	R^2	P	ASVs	R^2	P	ASVs
1	0.540	0.001	7318	0.422	0.001	6332	-	-	-
2	0.444	0.001	8322	0.321	0.001	7259	0.264	0.001	7930
3	0.404	0.001	7737	0.441	0.001	8097	0.412	0.001	8463
4	0.519	0.001	7183	0.506	0.001	6591	0.502	0.001	6698
5	0.456	0.001	7870	0.546	0.001	6807	0.557	0.001	7349
6	-	-	--	0.426	0.001	5456	0.618	0.001	6484
7	0.383	0.001	7932	0.518	0.001	7099	0.633	0.001	7922
8	0.519	0.016	6140	0.372	0.008	5924	0.546	0.008	5867
9	0.426	0.005	5750	0.484	0.010	5340	0.464	0.009	5216
10	0.371	0.007	5301	0.433	0.014	5928	-	-	-
11	0.526	0.001	7417	0.339	0.001	6879	0.351	0.001	6910
12	0.515	0.001	7235	0.475	0.001	7003	0.503	0.001	6512
13	0.339	0.001	7349	0.357	0.001	7128	-	-	-
14	0.517	0.001	7444	0.612	0.001	6943	0.524	0.001	6802
15	0.289	0.025	4821	0.345	0.008	5486	0.398	0.008	5397
16	0.498	0.001	7230	0.321	0.001	6694	-	-	-
17	0.557	0.001	7534	0.336	0.001	7110	0.502	0.001	7524
18	-	-	-	0.505	0.006	4753	0.529	0.013	4822
19	0.412	0.009	5587	0.436	0.010	5081	0.579	0.007	5336
20	0.560	0.001	5926	0.500	0.001	7253	0.483	0.001	5689
21	0.635	0.002	5872	0.618	0.001	6747	0.449	0.002	5623
22	0.327	0.012	5199	-	-	-	-	-	-
23	0.529	0.001	7725	0.566	0.001	8029	0.366	0.001	7198
24	0.560	0.001	7926	0.549	0.001	8085	0.576	0.001	6326

25	0.471	0.007	5870	0.434	0.012	5969	0.456	0.003	5675
26	0.556	0.011	5601	-	-	-	0.476	0.010	5122
27	0.589	0.009	5445	0.462	0.008	6037	0.545	0.006	5192
28	0.303	0.018	5637	0.372	0.006	6298	0.465	0.019	5258
29	0.416	0.009	5303	-	-	-	0.445	0.011	5129
30	0.494	0.012	5014	0.604	0.009	5248	0.554	0.013	4952
31	0.503	0.006	6048	0.499	0.007	5475	0.515	0.006	5478
32	0.533	0.010	3946	0.568	0.012	4379	0.477	0.014	4960
33	0.418	0.001	6412	0.397	0.011	6612	0.362	0.006	6217
34	0.496	0.008	5567	0.420	0.008	5793	0.476	0.032	4671
35	0.371	0.001	7050	0.358	0.001	7162	0.341	0.001	7590
36	0.385	0.002	7475	-	-	-	0.399	0.001	6890
37	0.461	0.001	8010	-	-	-	-	-	-
38	0.389	0.001	8222	-	-	-	0.420	0.001	8653
39	0.445	0.008	5761	0.461	0.007	5009	0.575	0.008	5643
40	0.570	0.001	6999	0.353	0.001	7369	0.480	0.001	6263
41	0.496	0.008	4590	0.484	0.100	4006	0.405	0.008	4449
42	0.397	0.011	6587	0.547	0.100	4851	0.480	0.013	5554
43	0.532	0.001	5620	-	-	-	0.468	0.004	5584
44	-	-	-	0.493	0.002	7418	0.674	0.002	7337

Supplementary Table 4. The list of software and algorithms used for each analysis.

Analysis methods	Software and algorithms	Source
Estimating of ecological processes	https://github.com/Pong2021/Agricultural-impacts-on-soil-microbiome-function.git	
Calculation of beta diversity	R package *vegan*	[1]
Linear mixed-effects model	R package *lme4*	[2]
DESeq2	R package *DESeq2*	[3]
SEM models	R package *lavaan*	[4]
FAPROTAX	FAPROTAX database	[5]
FungalTrait	FungalTrait database	[6]

Reference

1. Oksanen J*, et al.* Package 'vegan'. *Community ecology package, version* **2**, 1-295 (2013).

2. Bates D, Mächler M, Bolker B, Walker S. Fitting Linear Mixed-Effects Models Using lme4. *J Stat Softw* **67**, 48 (2015).

3. Love MI, Huber W, Anders S. Moderated estimation of fold change and dispersion for RNA-seq data with DESeq2. *Genome Biology* **15**, 550 (2014).

4. Rosseel Y. lavaan: An R Package for Structural Equation Modeling. *J Stat Softw* **48**, 36 (2012).

5. Louca S, Parfrey LW, Doebeli M. Decoupling function and taxonomy in the global ocean microbiome. *Science* **353**, 1272-1277 (2016).

6. Põlme S*, et al.* FungalTraits: a user-friendly traits database of fungi and fungus-like stramenopiles. *Fungal Diversity*, (2021).
