# Supplementary material for: Land conversion to agriculture induces taxonomic homogenization of soil microbial communities globally
Source: Nat Commun. 2024 Apr 29;15:3624. doi: 10.1038/s41467-024-47348-8 (PMC11058813; doi:10.1038/s41467-024-47348-8)
Supplement: Supplementary file 2 — Reporting Summary [file 41467_2024_47348_MOESM2_ESM.pdf]

nature portfolio

Corresponding author(s): Shuo Jiao, Gehong We

Last updated by author(s): Mar 13, 2024

Reporting Summary

Nature Portfolio wishes to improve the reproducibility of the work that we publish. This form provides structure for consistency and transparency in reporting. For further information on Nature Portfolio policies, see our Editorial Policies and the Editorial Policy Checklist.

Statistics

For all statistical analyses, confirm that the following items are present in the figure legend, table legend, main text, or Methods section.

n/a	Confirmed	
☐	☒	The exact sample size (*n*) for each experimental group/condition, given as a discrete number and unit of measurement
☐	☒	A statement on whether measurements were taken from distinct samples or whether the same sample was measured repeatedly
☐	☒	The statistical test(s) used AND whether they are one- or two-sided<br>*Only common tests should be described solely by name; describe more complex techniques in the Methods section.*
☐	☒	A description of all covariates tested
☐	☒	A description of any assumptions or corrections, such as tests of normality and adjustment for multiple comparisons
☐	☒	A full description of the statistical parameters including central tendency (e.g. means) or other basic estimates (e.g. regression coefficient) AND variation (e.g. standard deviation) or associated estimates of uncertainty (e.g. confidence intervals)
☐	☒	For null hypothesis testing, the test statistic (e.g. *F*, *t*, *r*) with confidence intervals, effect sizes, degrees of freedom and *P* value noted<br>*Give P values as exact values whenever suitable.*
☒	☐	For Bayesian analysis, information on the choice of priors and Markov chain Monte Carlo settings
☐	☒	For hierarchical and complex designs, identification of the appropriate level for tests and full reporting of outcomes
☐	☒	Estimates of effect sizes (e.g. Cohen's *d*, Pearson's *r*), indicating how they were calculated

Our web collection on statistics for biologists contains articles on many of the points above.

Software and code

Policy information about availability of computer code

Data collection	All statistical analyses were conducted in the statistical platform R (V4.2.1).
Data analysis	All scripts are available on GitHub (https://github.com/Pong2021/Agricultural-impacts-on-soil-microbiome-function.git) and Zenodo.

For manuscripts utilizing custom algorithms or software that are central to the research but not yet described in published literature, software must be made available to editors and reviewers. We strongly encourage code deposition in a community repository (e.g. GitHub). See the Nature Portfolio guidelines for submitting code & software for further information.

Data

Policy information about availability of data

All manuscripts must include a data availability statement. This statement should provide the following information, where applicable:

- Accession codes, unique identifiers, or web links for publicly available datasets
- A description of any restrictions on data availability
- For clinical datasets or third party data, please ensure that the statement adheres to our policy

All data required to reproduce the results are available in the Figshare Database (https://doi.org/10.6084/m9.figshare.25396525). The raw sequence data that support the findings of this study are openly available in the Beijing Institute of Genomics (BIG) Data Center, Chinese Academy of Sciences, under BioProject accession no. PRJCA020242 (16S amplicon) and PRJCA020245 (Metagenomics) and are publicly accessible at http://bigd.big.ac.cn/gsa. Source data are provided with this paper.

Research involving human participants, their data, or biological material

Policy information about studies with human participants or human data. See also policy information about sex, gender (identity/presentation), and sexual orientation and race, ethnicity and racism.

Reporting on sex and gender	n/a
Reporting on race, ethnicity, or other socially relevant groupings	n/a
Population characteristics	n/a
Recruitment	n/a
Ethics oversight	n/a

Note that full information on the approval of the study protocol must also be provided in the manuscript.

Field-specific reporting

Please select the one below that is the best fit for your research. If you are not sure, read the appropriate sections before making your selection.

☐ Life sciences ☐ Behavioural & social sciences ☒ Ecological, evolutionary & environmental sciences

For a reference copy of the document with all sections, see nature.com/documents/nr-reporting-summary-flat.pdf

Ecological, evolutionary & environmental sciences study design

All studies must disclose on these points even when the disclosure is negative.

Study description	We conducted a continental scale of 856 paired soils following agricultural conversion from natural ecosystems (forest, grassland, and wetland). A total of 1,185 soil samples were collected representing 856 paired soils, with 303 forest-cropland pairs, 275 grassland-cropland pairs, and 278 wetland-cropland pairs obtained (see Supplementary Table 2). A total of 2,403 samples were included in the global-scale meta-analysis.
Research sample	We analysed 16S sequences from 1185 soil samples from topsoil (20 cm depth) at continental scale and 2,403 topsoil samples at global scale. A subset of 40 samples from 10 regions covering cropland, forest, grassland and wetland soils at continental was selected for metagenomic sequencing to analyze changes in microbial community functional potential.
Sampling strategy	To reduce variation between regions as much as possible, we focused on fields planted with maize (Zea mays) to represent agricultural systems since maize is widely cultivated throughout China. Adjacent natural ecosystems were ~2 km from croplands and were selected to represent the most common and relatively undisturbed ecosystems, including forests, grasslands and wetlands. Among natural ecosystems of the 44 study regions, 30 regions include forests, grasslands, and wetlands, five regions include forests and wetlands, four regions include grasslands and wetlands, three regions include grasslands and wetlands, and two regions only include forests. In each region, we collected 4 to 10 plots in croplands based on field size.The other three natural ecosystems collected the similar number of soil samples corresponding to cropland. Composite surface soil samples (top ~20 cm depth) were collected at each plot.
Data collection	Soil samples were mixed by taking three soil cores with a 5-cm-diameter auger for each plot in the surface layer. After sampling, we thoroughly rinsed the soil auger using clean water. To ensure disinfection and sterilization, we then applied a 75% alcohol solution to its surface. Afterward, we placed the auger bit into a sterile bag for safe-keeping until the subsequent sampling event. These soil samples were sieved through a 2.0-mm mesh to remove plant roots, litter, rocks, and other debris. Team members labeled the samples using a permanent marker.
Timing and spatial scale	Sample collection of soils took place in croplands and adjacent natural ecosystems from 44 regions between July–August 2019 across China along the 3400 km latitudinal gradient from 23°39′ N to 47°79′ N and the 3200 km longitudinal gradient from 86°30′ E to 126°10′ E.
Data exclusions	No data were excluded from the analyses.
Reproducibility	We collected 4 to 10 plots as replicates of each ecosystem type in each region. Information about the sampled locations and methods used in this paper are included in our material and methods. All laboratory analyses were performed with two analytical replicates to ensure that data was reproducible. Different statistical tests were run at least three times to check consistency of findings.
Randomization	During all laboratory analyses, the samples were measured in a completely randomized fashion in order to avoid bias.
Blinding	Each sample was re-named by randomly assigning a number. This allowed all laboratory analyses to be conducted in a completely

randomized fashion, without knowledge of which sample was being analyzed.

Did the study involve field work? ☒ Yes ☐ No

Field work, collection and transport

Field conditions	We conducted a continental field survey in croplands and adjacent natural ecosystems from 44 regions across China. Among natural ecosystems of the 44 study regions, 30 regions include forests, grasslands, and wetlands, five regions include forests and wetlands, four regions include grasslands and wetlands, three regions include grasslands and wetlands, and two regions only include forests. The study survey represents a wide range of climate and soil gradients of climate, soil, and vegetation types (from tropical to boreal zones). For instance, mean annual precipitation and mean annual temperature in these regions are from 78 to 1775 mm and -2.8 to 24.4 ºC, respectively. Soil pH ranged from 4.63 to 10.18 and soil organic matter ranged from 4.64 to 60.22 g·kg−1 across all of the survey regions, representing broad environmental conditions.
Location	The study was conducted across China along the 3400km latitudinal gradient from 23°39′N to 47°79′N and the 3200km longitudinal gradient from 86°30′E to 126°10′E.
Access & import/export	Samples were collected following national rules. In some cases (private land) owners were duly informed upon collection. No permission was sought for land in public ownership.
Disturbance	There was minimal disturbance to the farmers' fields during this study, in which we only took no more than 10 soil samples from each site for each ecosystems in a 50 m radius. At each site, team members walked between rows and avoided trampling plants.

Reporting for specific materials, systems and methods

We require information from authors about some types of materials, experimental systems and methods used in many studies. Here, indicate whether each material, system or method listed is relevant to your study. If you are not sure if a list item applies to your research, read the appropriate section before selecting a response.

Materials & experimental systems

n/a	Involved in the study	
☒	☐	Antibodies
☒	☐	Eukaryotic cell lines
☒	☐	Palaeontology and archaeology
☒	☐	Animals and other organisms
☒	☐	Clinical data
☒	☐	Dual use research of concern
☒	☐	Plants

Methods

n/a	Involved in the study	
☒	☐	ChIP-seq
☒	☐	Flow cytometry
☒	☐	MRI-based neuroimaging
